# Homeostatic Maintenance of Allele-Specific *p16* Methylation in Cancer Cells Accompanied by Dynamic Focal Methylation and Hydroxymethylation

**DOI:** 10.1371/journal.pone.0097785

**Published:** 2014-05-14

**Authors:** Sisi Qin, Qiang Li, Jing Zhou, Zhao-jun Liu, Na Su, James Wilson, Zhe-ming Lu, Dajun Deng

**Affiliations:** 1 Key Laboratory of Carcinogenesis and Translational Research (Ministry of Education), Division of Cancer Etiology, Peking University Cancer Hospital & Institute, Beijing, China; 2 GRU Cancer Center, Georgia Regents University, Augusta, Georgia, United States of America; Northwestern University, United States of America

## Abstract

**Aim:**

*p16* Methylation frequently occurs in carcinogenesis. While it has been hypothesized that the *p16* methylation states are dynamically maintained in cancer cells, direct evidence supporting this hypothesis has not been available until now.

**Methods:**

A fusion cell model was established which reprogrammed the native DNA methylation pattern of the cells. The methylation status of the *p16* alleles was then repeatedly quantitatively analyzed in the fusion monoclonal, parental cancer cell lines (*p16*-completely methylated-AGS and unmethylated-MGC803), and HCT116 non-fusion cell using DHPLC and bisulfite sequencing. Histone methylation was analyzed using chromatin immuno-precipitation (ChIP)-PCR. P16 expression status was determined using immuno-staining and RT-PCR.

**Results:**

The methylation status for the majority of the *p16* alleles was stably maintained in the fusion monoclonal cells after up to 60 passages. Most importantly, focal de novo methylation, demethylation, and hydroxymethylation were consistently observed within about 27% of the *p16* alleles in the fusion monoclones, but not the homozygously methylated or unmethylated parental cells. Furthermore, subclones of the monoclones consistently maintained the same *p16* methylation pattern. A similar phenomenon was also observed using the *p16* hemi-methylated HCT116 non-fusion cancer cell line. Interestingly, transcription was not observed in *p16* alleles that were hydroxymethylated with an antisense-strand-specific pattern. Also, the levels of H3K9 and H3K4 trimethylation in the fusion cells were found to be slightly lower than the parental AGS and MGC803 cells, respectively.

**Conclusion:**

The present study provides the first direct evidence confirming that the methylation states of *p16* CpG islands is not only homeostatically maintained, but also accompanied by a dynamic process of transient focal methylation, demethylation, and hydroxymethylation in cancer cells.

## Introduction

DNA methylation is considered to be one of the most stable epigenetic modifications in mammals. The methylation states of cell differentiation related genes have been shown to be highly dynamic during germ cell and preimplantation development, but become relatively static during the development of somatic tissues [1]–[3]. In contrast, the methylation states of host adaptation related genes remain dynamic in somatic tissues in order to allow for the proper response to environmental factor exposure and subsequent pathogenesis [4]–[6]. Hydroxymethylation of DNA is intimately involved in altering gene methylation status and has been found to not only play a key role in DNA demethylation, but also serve many of its own functions [7], [8].

Tumor suppressor P16 encoded by the ink4/arf locus within human chromosome 9p21 is a cell cycle regulator involved in the inhibition of G1 → S phase transition through the P16-CDK4/6-RB pathway [9]. The locus is transcriptionally silenced in embryonic stem cells, and plays a rate-limiting role in the reprogramming of induced pluripotent cells [10]. Although a 9p21 fragment deletion is the most frequent genetic alteration found in all cancers [11], hypermethylation of CpG islands is still the main mechanism for p16 inactivation in multiple human cancers [12]–[14]. In fact, a number of cohort studies have shown that p16 methylation occurs early in carcinogenesis and has been shown to significantly increase the risk of malignant transformation of epithelial dysplasia [15]–[21]. Even though the causative role of p16 methylation in carcinogenesis has not been established, the evidence strongly suggests that p16 methylation may contribute significantly to cancer development and could be developed into a potential biomarker [4]. Although p16 methylation is one of the most highly studied epigenetic modifications, its stability in cancer cells and the mechanism through which it is maintained has not yet been clarified [22]–[26]. A detailed understanding of the maintenance machinery involved in DNA methylation is a critical step for the development of methylation biomarkers and epigenetic intervention therapy.

The prevalence of p16 methylation in human chronic gastritis tissues is strongly correlated with Helicobacter pylori infection, and dramatically decreased after H. pylori eradiation, suggesting that most methylated-p16 alleles may not be stable [27], [28]. In contrast, p16 methylation in cultured normal and cancer cells is likely very stable as it was efficiently recovered after the DNA methylation inhibitor (5-aza-CdR) was removed [29]. However, reactivation of methylation-silenced genes was observed in a certain proportion of the DNA methyltransferase inhibitor-treated cells. It is difficult to exclude the possibility that the observed recovery of p16 methylation results from a selection advantage conferred to cells that do not undergo p16 reactivation/demethylation during the inhibitor treatment. In addition, it has recently been reported that methylated CpG sites within the p16 CpG islands are mainly located in the p16 exon-1 coding nucleosomal region in human gastritis lesions and extend into the 5′UTR and promoter nucleosomal regions during the development of gastric carcinomas [26]. These findings suggest that the fully methylated-p16 alleles might be more stable than the focally methylated alleles; however, this hypothesis should be further tested using monoclonal cells containing p16 alleles with diverse methylation patterns, including focal methylation.

Cell fusion has been used to study the mechanisms behind gene transcription regulation and DNA methylation alteration [24], [30]. In order to establish a cell model containing diverse p16 methylation patterns, a gastric cancer cell line with completely methylated p16 (AGS) was combined with a p16-active gastric cancer cell line (MGC803) through cell fusion. Next, these fusion cells were cloned and subcloned before the distribution of methylated and hydroxymethylated CpG sites within the p16 CpG islands was characterized. The methylation status of most p16 alleles in the fusion cells remained the same as the parental cells. Low levels of focal de novo methylation, demethylation, and hydroxymethylation were also observed in the fusion cells; however, these changes were found to be instable events. A similar phenomenon was also observed using the p16 hemi-methylated cell line HCT116. These findings indicate that the methylation states of p16 CpG islands are homeostatically maintained in cancer cells. To the best of our knowledge, this is the first report to suggest that the methylation status of CpG islands is homeostatically maintained in cancer cells not undergoing differentiation.

## Materials and Methods

### Cell culture

Human gastric cancer cell line MGC803 was cultured in RPMI 1640 medium with 10% FBS. AGS was cultured in F12 medium with 10% FBS. MGC803 [31] and AGS (ATCC CRL-1739) cell lines were kindly provided by Prof. Yang Ke at Peking University Cancer Hospital and Institute. HCT116 cells were kindly provided by Dr. Yuanjia Chen, Peking Union Hospital (ATCC CCL-247), and were cultured in RPMI 1640 medium with 10% FBS.

### Generation of the stable fusion cell clones

pBabe-puro (hygromycin resistance) and pIRES2-EGFP (neomycin/G418 resistance) vectors were transfected to AGS and MGC803 cells, respectively, using LipofectamineTM 2000 (Invitrogen, 11668-027) according to the manufacturer's instruction. Puromycin (puromycin^r+^-AGS) and G418 (neomycin^r+^-MGC803) selection was performed on AGS and MGC803 cell lines followed by culture in F12 medium with 10% FBS and puromycin (0.25 µg/mL) and RPMI 1640 medium with 10% FBS and neomycin (700 µg/mL), respectively. The fusion was performed using PEG8000 as described previously [32]. After fusion, the cells were cultured in selection medium containing both neomycin (700 µg/mL) and puromycin (0.25 µg/mL). Following selection, individual colonies were mono-cloned, and after three rounds of subsequent cloning, five monoclones were obtained. Two of the five monoclones were chosen for further mono-cloning.

### Microsatellites *D9S974* and *D9S1749* analysis

The primer set for *D9S974* (sense, 5′-gagcc tggtc tggat cataa-3′; antisense, 5′-aagct tacag aacca gacag-3′) and *D9S1749* (sense, 5′-aggag agggt acgct tgcaa-3′; antisense, 5′-tacag ggtgc gggtg cagat aa-3′) were used to amplify the *p16* alleles (Figure S1A). Genotypes of the micorsatellites were analyzed at 80°C by DHPLC using a UV detector (260 nm) as described previously [33].

### Karyotype analysis

A 25-cm flask at 60% cell confluence was treated with 0.2 µg/mL colchicine for 4 hrs. Cells were recovered after trypsinization and treatment with 75 mmol/L KCl solution for 25 min. The cells were fixed (3∶1 methanol: acetic acid), and stained with Giemsa stain.

### DNA preparation, bisulfite and TAB treatment

Genomic DNA was extracted from tissue samples with phenol/chloroform. The samples were treated with 5 mol/L of sodium bisulfite for 16 hrs at 50°C [34]. For 5hmC analysis, genomic DNA was treated using the TAB Kit according to manufacturer's specifications (WiseGene, Cat# K001) [35].

### Hydroxymethylated DNA-specific immunoprecipitation (hMeDIP)

Genomic DNA was sonicated into 200 bp to 600 bp fragments using Bioruptor sonicator (Diagenode). 100 ng sonicated DNA was saved as the input control. Each sample consisted of 1 µg sonicated DNA diluted in IP buffer and incubated with 4 µl anti-hydroxymethylcytosine (5hmC) antibody (Active Motif) at 4°C overnight. The antibody-DNA complexes were captured using protein A/G beads then purified. PCR was run on all samples using 35 cycles with the primers 5′-agtcct ccttc cttgc caac-3′ and 5′-tccga gcact tagcg aatgt g-3′. Unmethylated cytosine, 5-methylcytosine, and 5hmC DNA fragments were used as PCR controls to verify 5hmC enrichment.

### Chromatin-immunoprecipitation (ChIP) assays

Levels of histone modifications H3K9me3 and H3K4me3 were determined using ChIP assays as described [36].

### PCR amplifications

The 392 bp amplicon of the antisense-strand of the *p16* exon-1 was amplified with the CpG-free primer set (sense, 5′-ttttt agagg atttg aggga tagg-3′; antisense, 5′-ctacc taatt ccaat tcccc tacaa acttc-3′) as described [37]. The 588 bp amplicon of the antisense-strand of the *p16* exon-1 and promoter was amplified using the sMSP primer set (sense, 5′-ctacct aattc caatt cccct aca-3′; antisenses, 5′-ccaat tcccc tacaa acttc g-3′, 5′-ccaat tcccc tacaa acttc atcct ccaaa atcg-3′ and 5′-ccaat tcccc tacaa acttc atcct ccaaa atcac ccg-3′) [26]. The 369 bp of the sense-strand of the *p16* exon-1 was amplified with the CpG-free primer set (sense, 5′-gttgt agatt tttta tttat ttgga t-3′; antisense, 5′-tcccc ttacc taaaa aaata cc-3′) at annealing temperature 56°C. The 284 bp and 346 bp amplicons of the antisense-strands of the *p16* exon-2 and intron-2 were respectively amplified with the CpG-free primer set (sense, 5′-tggta ggtta tgatg atgggt ag-3′; antisense, 5′-aacat aatta ctacc tctaa taccc c-3′) and (sense, 5′-tttgt gggtt tgtag aagta ggtat g-3′; antisense, 5′-cctaa tccaa caact aatcc actac c-3′) at annealing temperature 57°C.

### Methylation quantification of CpG islands using DHPLC

The 392 bp, 369 bp, 284 bp, 346 bp PCR products amplified by universal primers were separated using the WAVE DNA Fragment Analysis System with a fluorescence (FL)-detector (Transgenomic, Inc., OM, USA) at 57.6, 55.0, 58.0, and 57°C, respectively [37], [38]. The WAVE-HS1 FL-dye buffer (Transgenomic, Inc.) was used to enhance the FL-intensity of PCR products (universal post-column labelling). Genomic HCT116 DNA was used as standard control.

### Clone sequencing

PCR was performed on the bisulfite converted DNA. The amplicons were cloned into the pCR-blunt vector (Invitrogen), transformed into *E. coli*, and sequenced using an ABI 3730 Analyzer.

### *p16* RT-PCR

*p16* mRNA was detected as described [12].

### P16 immunofluorescence staining

Immunofluorescence staining was performed as previously described [39]. In short, fusion cells were fixed in 4% formaldehyde/PBS for 10 min before a 5-min wash in PBS. Fixed samples were blocked for 30 min with 0.5% Triton X-100/PBS at room temperature. Cells were incubated with anti-P16 antibody (Abcam, ab54210) for 1 h at 37°C, after washed with 0.5% Triton X-100/PBS, then incubated with goat-anti rabbit IgG antibody labeled with rhodamine for 1 h at 37°C. DAPI (1∶2000) was used to stain the cell nucleus. Photos were taken on a Leica confocal microscope.

## Results

### Establishment and characterization of a cell fusion model with diversely methylated *p16* alleles

It has been reported that heterokaryons can induce DNA methylation variation via cell fusion of two cell lines [30]. We hypothesized that a cell fusion model containing different methylation states of p16 CpG islands could duplicate the de novo methylation or demethylation processes. Thus, puromycin^r+^-AGS cells containing fully methylated-p16 alleles and the neomycin^r+^-MGC803 cells containing unmethylated-p16 alleles were constructed, respectively. These two cell lines were then fused and cultured in selection medium containing both puromycin and neomycin. After three rounds of cloning, the fusion monoclones were characterized using two microsatellites (D9S974 and D9S1749) near the p16 locus (Figure S1A). Two D9S1749 microsatellite chromatogram patterns (C7/C9/E10 and D3/E3) and one D9S974 pattern were observed in 5 tested monoclones that differed from the patterns of their parental cells. Chromosome mode was also different between the representative clone E10 (near-tetraploid) and its parental cells (near-diploid) (Figure S1B).

### Stable maintenance of completely methylated and unmethylated-*p16* alleles in cancer cells

**Figure 1:**
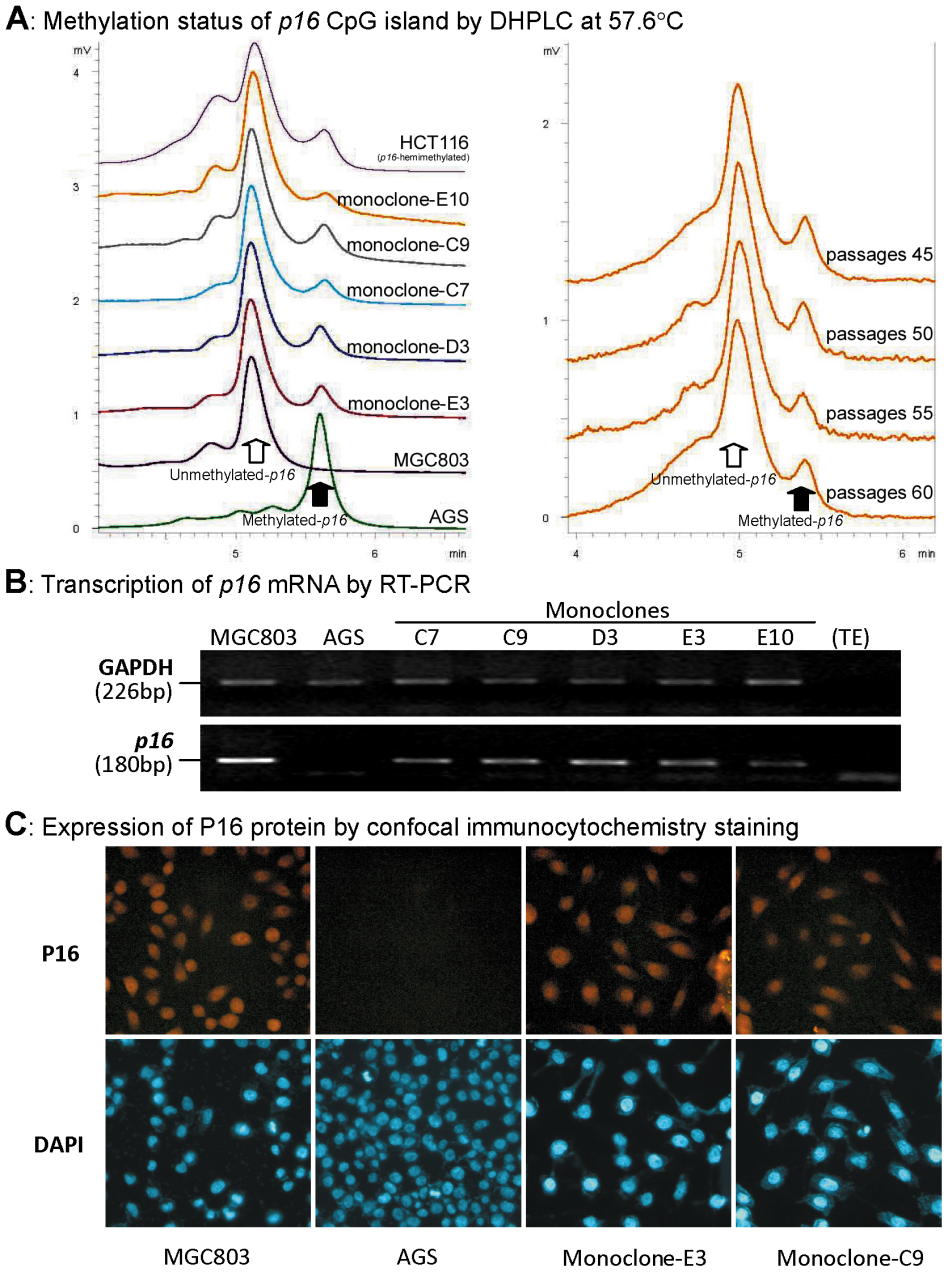
Characterization of methylation and expression of *p16* in the fusion cell lines. (**A**) The methylation states of *p16* CpG islands in the fusion monoclones were analyzed using DHPLC (left). The proportion of methylated-*p16* in the monoclone-E10 was stably maintained at passages 45, 50, 55, and 60 (right). (**B**) RT-PCR shows varying mRNA expression of *p16* in the fusion monoclones and their parental MGC803 and AGS cell lines. (**C**) Confocal immunocytochemistry staining shows varying P16 expression in the various cell lines.

The methylation status of the p16 CpG islands (392 bp) was quantitatively analyzed in these fusion clones using a DHPLC assay as previously reported [37]. As was seen in the p16 hemi-methylated HCT116 cells, the ratio of methylated- to unmethylated-p16 molecules was about 1∶1 in these fusion clones (Figure 1A-left). The proportion of methylated-p16 alleles was stably maintained in a representative clone from passages 45, 50, 55, and 60 (Figure 1A-right). Furthermore, RT-PCR detected p16 mRNA in all 5 tested clones (Figure 1B). Nucleoplasmic P16 protein was also observed within each cell in two representative monoclones (C9 and E3) (Figure 1C). Based on this information, it is evident that the transcription/methylation states of the p16 alleles in the fusion cells is likely maintained in a similar pattern to their parental cells.

### Focally methylated *p16* alleles are not stable in cancer cells

**Figure 2:**
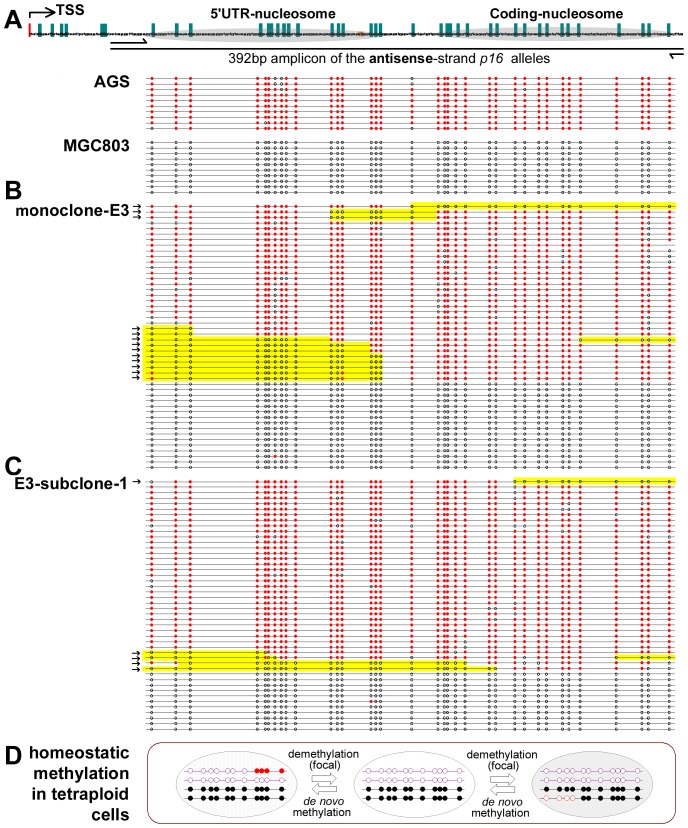
Bisulfite sequencing of *p16* alleles in the fusion cells and their parental cells. (**A**) Bisulfite clone sequencing of the 392 bp fragment of the *p16* CpG island in AGS and MGC803 cells; Each green bar represents a CpG site. Nucleosomal location in exon-1 is also marked (26). (**B** and **C**) Bisulfite sequencing in the fusion monoclone-E3 and subclones. Focal methylation changes (yellow-shadowed); methylated CpG sites (red dot); unmethylated CpG sites (open dot); focally methylated- or demethylated-*p16* molecules (black arrows). (**D**) Homeostatic methylation model for a single CpG island in the tetraploid cells.

Interestingly, while most p16 molecules remained in the completely methylated or unmethylated states, the representative fusion clones contained about 27% (13/48) focally methylated- (or demethylated-) p16 molecules in the exon-1 region (Figure 2B). However, the homozygously methylated or unmethylated parental cells did not exhibit the same methylation changes. In order to confirm that the tested clone was in fact monoclonal, this clone was further subcloned. Further analysis revealed that the p16 methylation patterns in two subclones consistently remained similar to their parental clones (Figure 2C and Figure S2).

A single nucleotide polymorphism (SNP) (rs36228836) was identified in the p16 promoter region. It was determined that the genotype of this SNP differed between the two parental cell lines (T/**A** for MGC803 and T/T for AGS), thus making it possible to identify the MGC803-specific p16 **A**-alleles in the fusion cells (Figure S3). In order to clarify whether these focally methylated fragments were the result of de novo methylation or demethylation, the methylation states within a 588 bp fragment covering this SNP were analyzed using a seeding methylation-specific PCR assay (sMSP). It was discovered that CpG methylation of the p16 alleles at the methylation “seeding” sites within intron-1 were effectively enriched [26]. Results of bisulfite-sequencing confirmed that focal de novo methylation was observed in the MGC803-specific **A**-alleles of monoclone-D3 (Figure S3).

**Figure 3:**
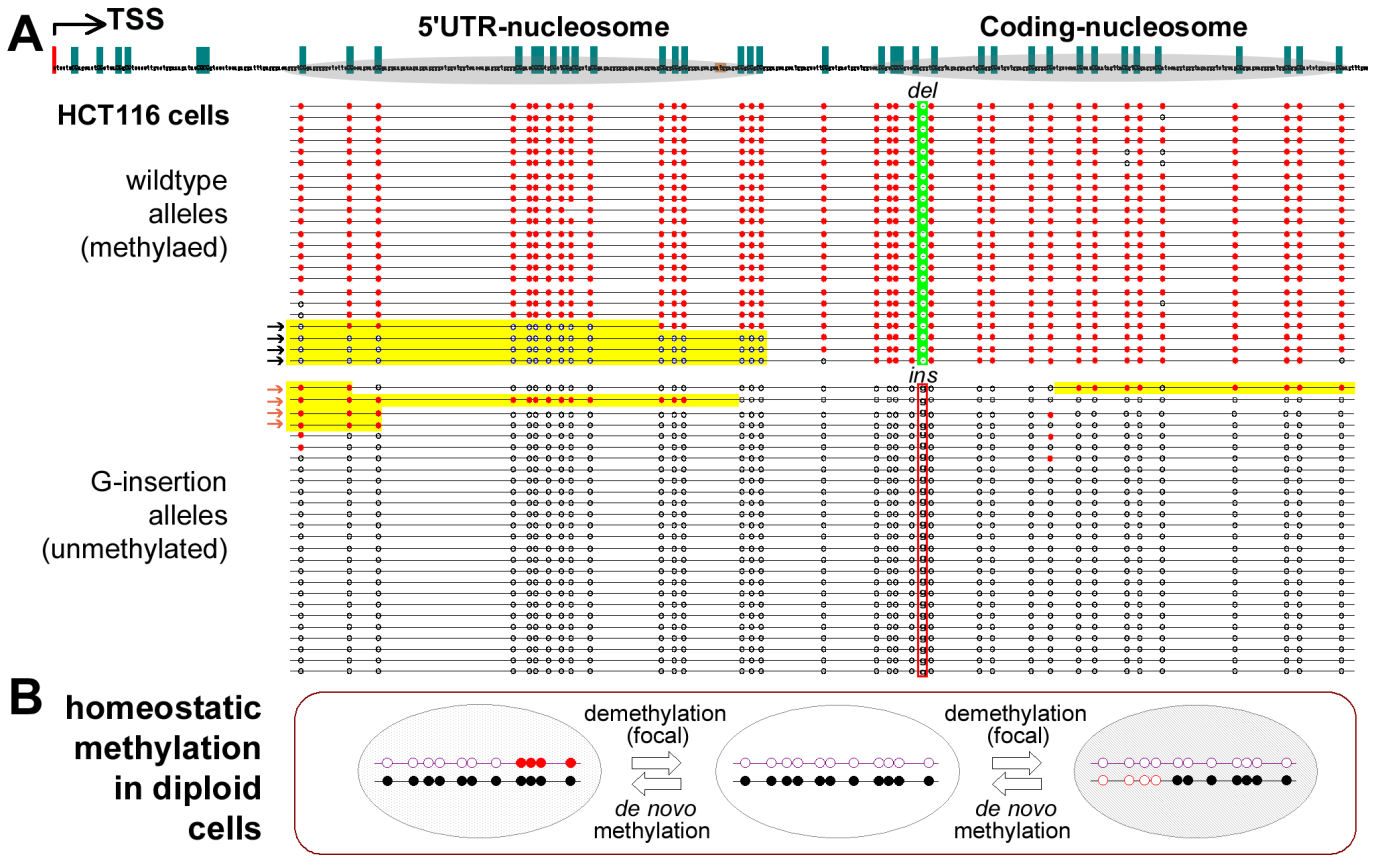
Bisulfite sequencing of *p16* alleles in HCT116 cells. (**A**) Bisulfite clone sequencing of the 392 bp fragment of the *p16* CpG island in HCT116 cells. Wild-type (*deletion*)/mutant (G-*insertion*) *p16* alleles; focally demethylated-*p16* molecules (black arrows); focally *de novo* methylated molecules (red arrows). (**B**) Homeostatic methylation model for a single CpG island in the diploid cells.

In order to investigate whether the transient focally methylated-p16 alleles exist in non-fusion cells, the methylation pattern of the p16 hemi-methylated HCT116 cell line was also analyzed. Similarly, 16% (8/49) of the p16 molecules showed focal methylation in the HCT116 cells (Figure 3). Furthermore, by using a del/ins reading frame-shift mutation found in the exon-1 coding region as a genetic marker, it was determined that both focal de novo methylation in the mutant G-insertion alleles (mainly unmethylated) and demethylation in the wild-type alleles (mainly methylated) also occur sporadically in the HCT116 cells (4/26 and 4/23, respectively). This indicates that the focal methylation and demethylation of p16 alleles do in fact exist in non-fusion cancer cells. Taken together, these results suggest that while focal methylation and demethylation are relatively common events, most of the completely methylated- and unmethylated-p16 alleles are stably maintained in cancer cells.

In order to study whether the focal methylation/demethylation occurs in the gene body, we analyzed the methylation states of CpG islands within the p16 exon-2 and intron-2. DHPLC analysis revealed that these two CpG islands were fully methylated in AGS, MGC803, and HCT116 cells (Figure S4A&B), and bisulfite-sequencing of the intron-2 CpG island confirmed the DHPLC results (Figure S4C), suggesting the gene body is homogenously and stably methylated in these cells and is not accompanied focal methylation/demethylation.

### Concomitant 5-hydroxymethylation occurs in the *p16* alleles of fusion cells

**Figure 4:**
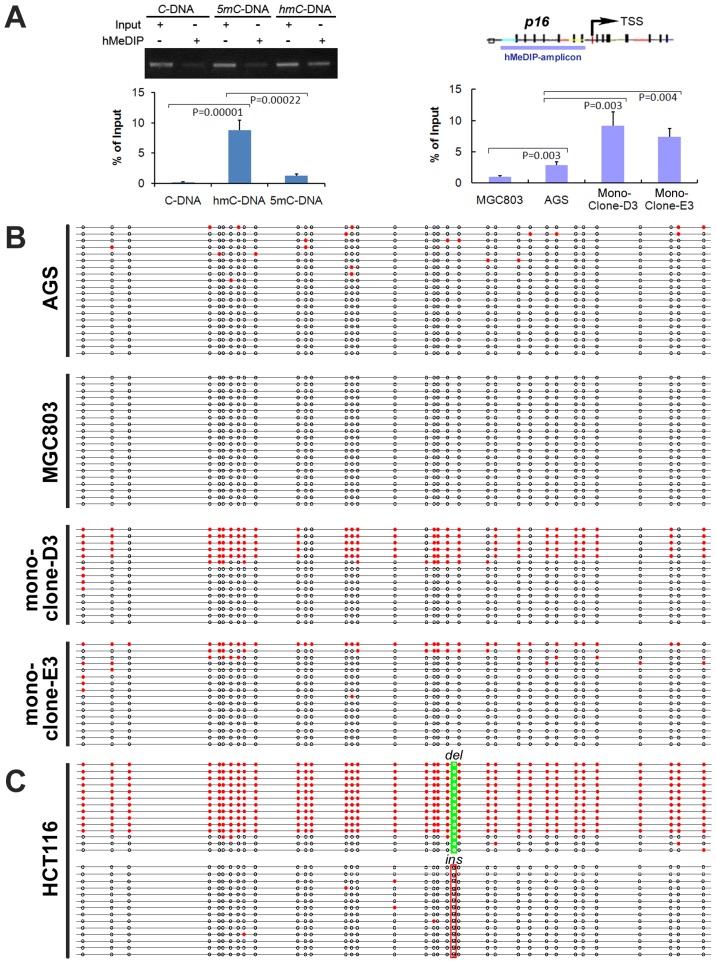
*p16* chromatin hydroxymethylation characterization. (**A**) Immuno-precipitation and PCR analysis of synthetic and cellular 5hmC-containing *p16* sequences. The AGS and fusion monoclone-D3 and E3 cells show significant levels of 5hmC. (**B**) TAB-seq analysis reveals completely hydroxymethylated-*p16* alleles in the fusion subclones, but not the parental cells. (**C**) TAB-seq analysis of HCT116 cells shows the majority of the wildtype *p16* alleles are completely hydroxymethylated.

DNA methylation and hydroxymethylation cannot be readily differentiated using regular bisulfite-based methylation assays. The role of hydroxymethylation in the focal p16 methylation or demethylation was investigated using hMeDIP-PCR assay (Figure 4A, left chart). The results of this assay showed that the amount of hydroxymethylated-p16 DNA in the two fusion clones was significantly higher than their parent cells (Figure 4A, right chart).

Tet-assistant bisulfite sequencing (TAB-seq) is capable of specifically detecting 5-hydroxy- methylcytosine (5hmC) in DNA at a single base resolution [35]. Using this assay to analyze the 5hmC content in the p16 CpG islands in details, it was found that AGS cells exhibit sporadic hydroxymethylated CpGs at a very low frequency (21/700), while the MGC803 cells did not show any hydroxymethylation. As expected, both complete and focal hydroxymethylation of p16 alleles were detected in these fusion cells (Figure 4B). The results of two assays consistently indicate that hydroxymethylation may play a role in the homeostatic maintenance of p16 methylation in the fusion cells.

**Figure 5:**
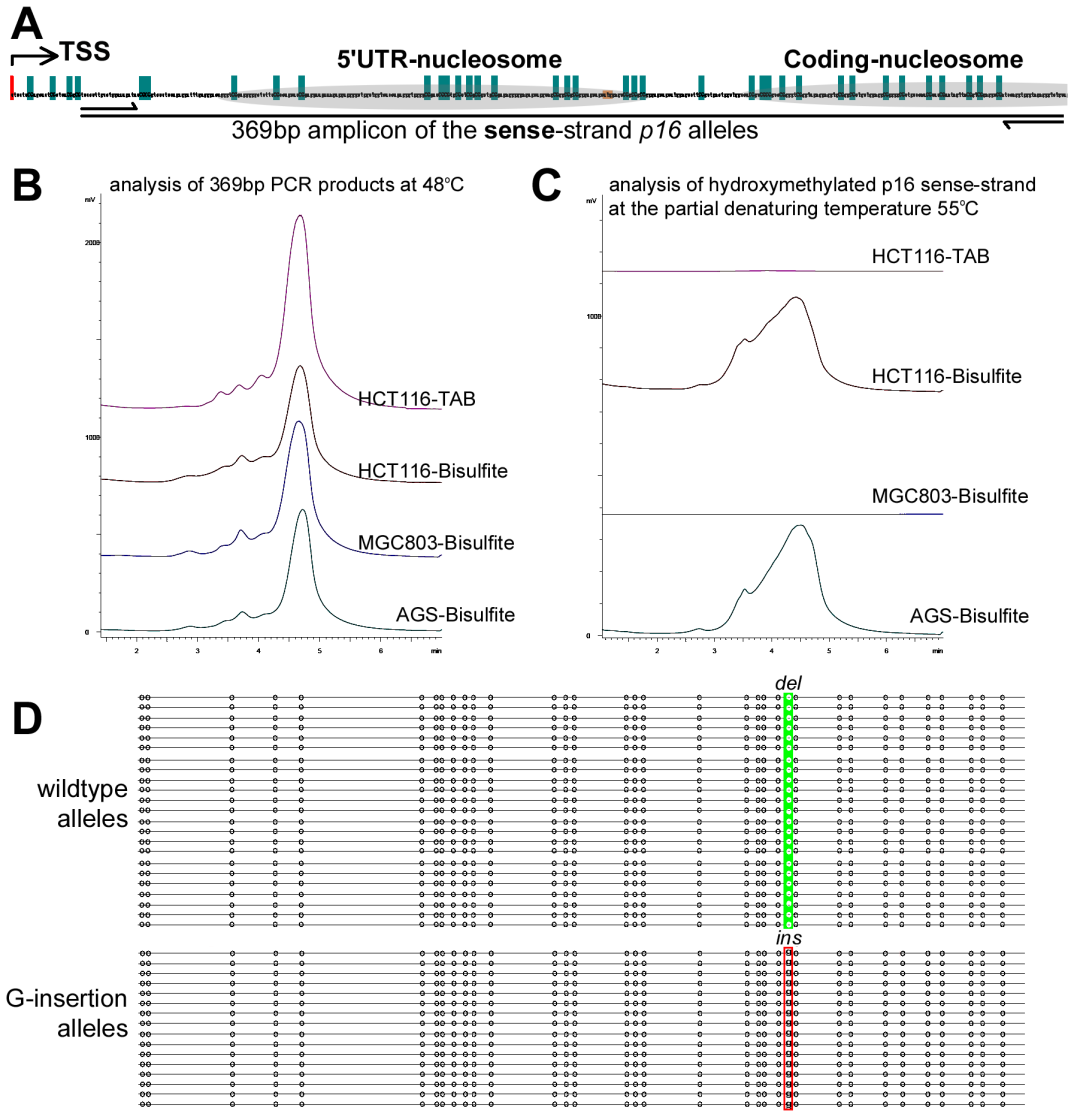
Analysis of hydroxymethylation in the sense-strand of *p16* CpG island in HCT116 cells. (**A**) Location of the 369 bp amplicon of the sense-strand *p16* exon-1. (**B**) Detection of the 369 bp PCR products using DHPLC (sizing mode) at 48°C. (**C**) Detection of hydroxymethylated molecules amplified from the *p16* sense-strand after TAB modification using DHPLC (FL detector) at the partial denaturing temperature 55°C. Under this condition, the hydroxymethylated- and unhydroxymethylated-*p16* molecules are partially and completely denatured, respectively. The regular bisulfite-treated templates are used as reference controls. (**D**) Results of TAB-sequencing for the 369 bp PCR products amplified from HCT116 cells.

Analysis of HCT116 cells unexpectedly revealed frequent and complete hydroxymethylation within the antisense-strands of the p16 wildtype (del) alleles (11/14), but not in the G-insertion mutant alleles (Figure 4C). While methylation was detected in the 369 bp fragment of the sense-strand of the p16 exon-1 in HCT116 cells as expected (Figure 5A–C), notably, the complete hydroxymethylation was not observed in the sense-strands in both TAB-DHPLC analysis and TAB-sequencing (Figure 5C and D), suggesting the antisense strand specific hydroxymethylation.

**Figure 6:**
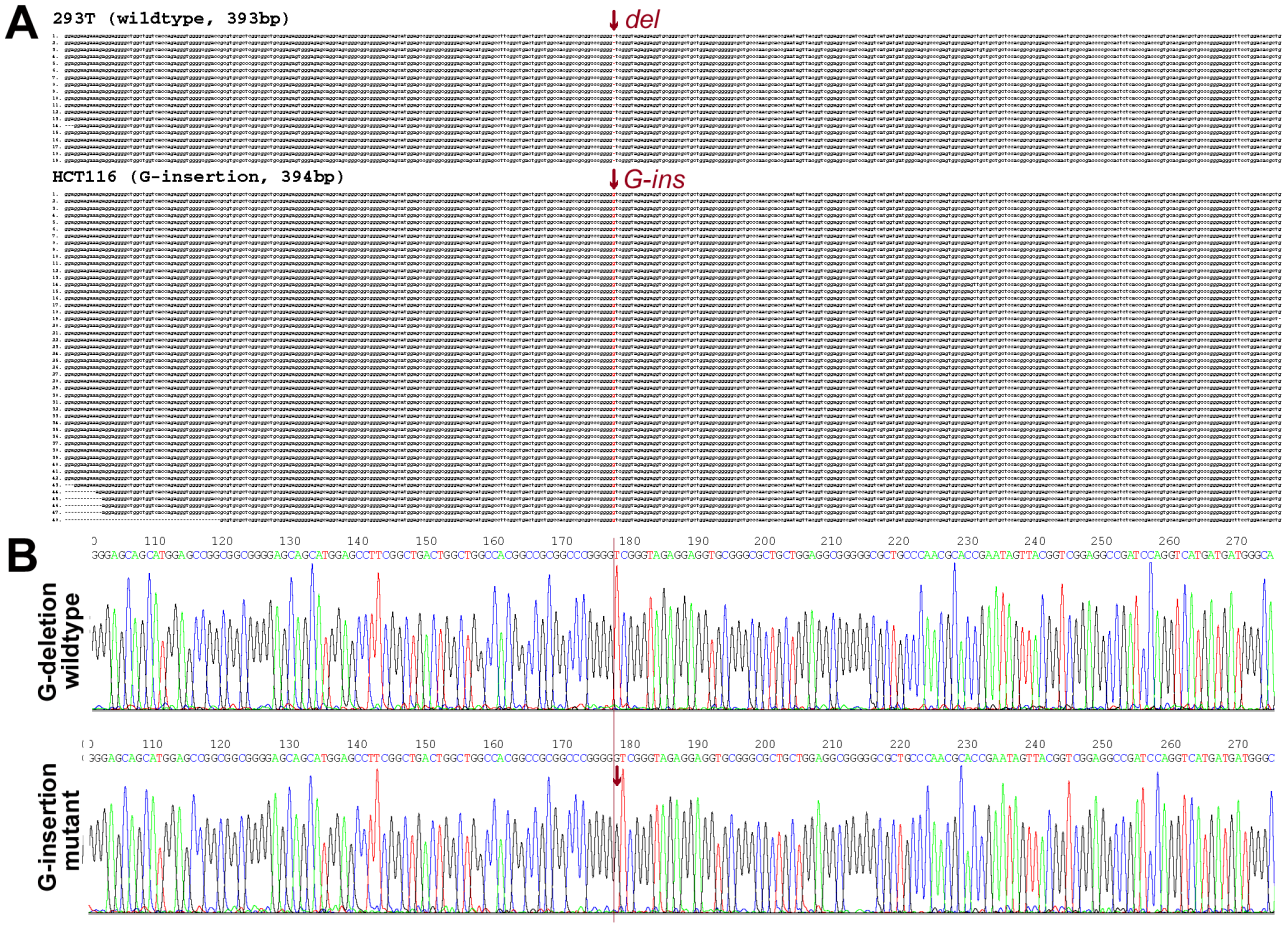
Allele specific RT-PCR analysis of *p16* mRNA of cells. (**A**) RT-PCR clone sequencing of the control 293T cell line (del) shows wildtype *p16* mRNA. The HCT116 (G-ins) cell line revealed only mutant *p16* mRNA clones. (**B**) Clone sequencing results show both wildtype and mutant p16 mRNA.

In order to determine if the completely hydroxymethylated wildtype p16 alleles are transcriptionally active, the allele-origin of p16 transcripts was characterized by clone sequencing. Results of the sequencing revealed that none of the 48 p16 mRNA molecules were transcribed from wildtype p16 alleles that were asymmetrically methylated and hydroxymethylated (M∶H) (Figure 6). This information proves that hydroxymethylation in the antisense strand of the p16 alleles may not lead to transcriptional activation of the gene.

### Concomitant of histone H3K4 and H3K9 trimethylation at the *p16* alleles in fusion cells

**Figure 7:**
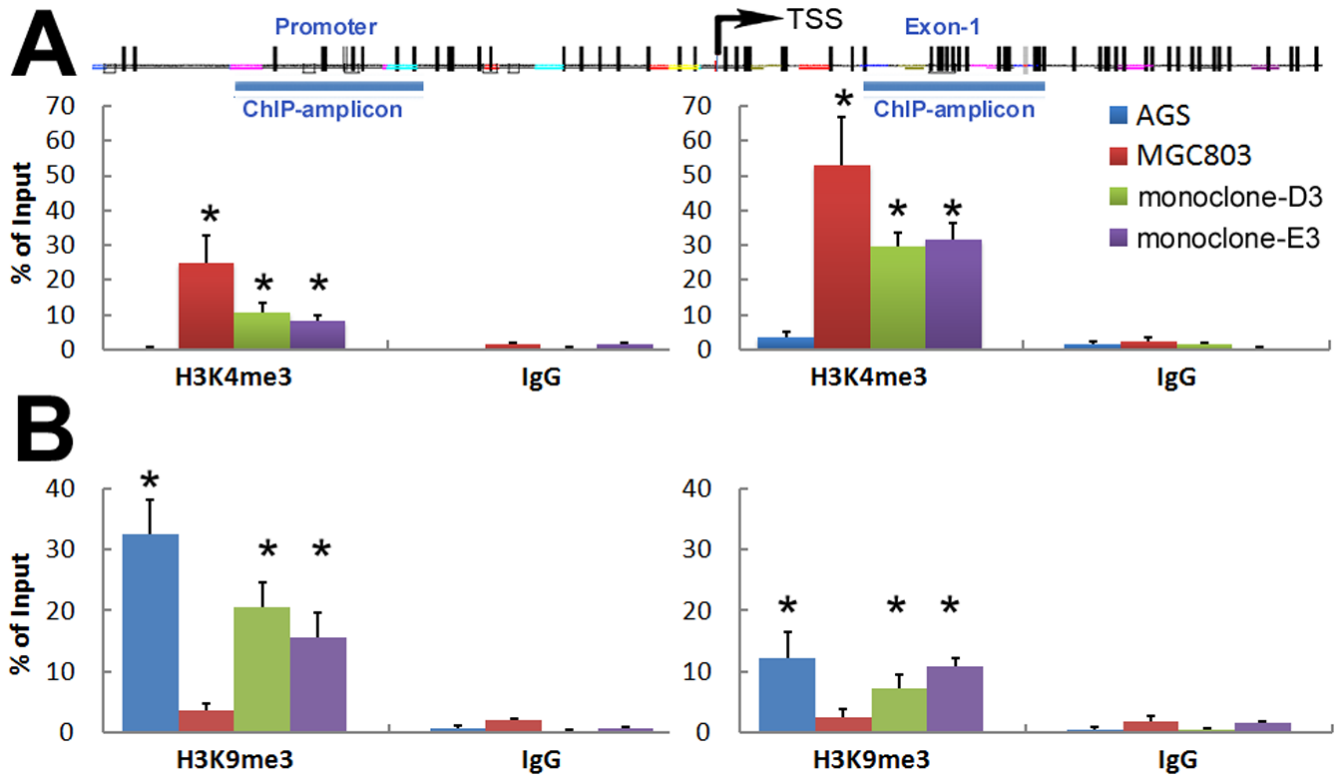
Characterization of histone modifications in the *p16* chromatin by ChIP-PCR assays. (**A**) The active H3K4me3 level within the *p16* CpG islands in the fusion subclones and MGC803 cells was significantly higher than AGS cells, especially in the exon-1 region (monoclones vs. AGS, P<0.01). (**B**) The repressive H3K9me3 level in the fusion subclones and AGS cells was significantly higher than MGC803 cells, especially in the promoter region (monoclones vs. MGC803, P<0.01).

ChIP-PCR was used to quantitatively determine the levels of active H3K4me3 and repressive H3K9me3 in the fusion cells (Figure 7). The H3K4me3 level in the heterogeneously methylated p16 alleles of the fusion subclones was found to be significantly lower than was seen in the completely unmethylated alleles of the MGC803 cells. Furthermore, the level of H3K4me3 in exon-1 chromatin was determined to be higher than was found in promoter chromatin. While the H3K4me3 was not detected in the AGS cells, the H3K9me3 level was significantly higher in this cell line than other cell lines. H3K9me3 level showed an inverse correlation with the H3K4me3 level among these cell lines.

## Discussion

The DNA methylation pattern is known to change dynamically during the differentiation of embryonic stem cells and development of tissues in vertebrates. However, it remains a mystery how the methylation states of the genome are maintained in cells no longer undergoing differentiation. In the present study, it was found that the methylation states of completely methylated- and unmethylated-*p16* alleles remained stable during cell proliferation. Furthermore, low levels of focal demethylation, hydroxymethylation, and *de novo* methylation occurred relatively frequently in two kinds of *p16* hemi-methylated cancer cell models, but not in homogeneously methylated or unmethylated cells. These findings imply that homogeneous methylation can exert repression pressure on the *p16* gene that can at least partially affect formerly unmethylated CpG islands. In contrast, the transcription pressure exerted by complete demethylation of the gene is capable of inducing and maintaining demethylation of *p16* CpG islands. Hydroxymethylation of *p16* CpG alleles was also discovered to play a role in the homeostatic maintenance of genome methylation. To our knowledge, this is the first report to show that the methylation status of a tumor suppressor gene is homeostatically maintained in cancer cells.

The stability of methylated p16 alleles has not yet been explained. In gastritis lesions, the methylation states of most p16 alleles are unstable and H. pylori-dependent [27], [28]; however, in cancer cell lines they have proven to remain remarkably stable [29]. Our recent comprehensive sequencing studies revealed that the methylated CpG sites in gastritis are located within the exon-1 region of the p16 gene, but extend into the promoter region after the development of gastric carcinoma. This implies that completely methylated-p16 alleles are considerably more stable than their focally methylated counterparts [26]. It has also been reported that heterokaryons can induce DNA methylation variations via cell fusion of two cell lines, and furthermore that some silenced-p16 molecules in mouse cancer cells can be reactivated after being fused with p16-active mouse embryonic stem cells [30], [39]. In the present study, fusion cells containing a wide variety of p16 methylation patterns were established from homozygously methylated and unmethylated parental cell lines in order to investigate the stability of focally and completely methylated-p16 alleles within the same cell. As expected, the p16 alleles of the fusion cells showed a complete and variable range of methylation. The p16 alleles in the fusion clones and their subclones remained stably hemi-methylated during cell passages, which indicate that these completely methylated and unmethylated p16 alleles are relatively stable. On the other hand, hemi-methylated fusion clones and HCT116 cells exhibited low levels of focally methylated/demethylated-p16 alleles, which provide direct evidence that the hemi-methylated p16 alleles are less stable than the homogenously methylated p16 alleles. This dynamic, but still homeostatic, methylation pattern of p16 CpG islands may offer a common mechanism explaining DNA methylation maintenance in differentiation static cells. In addition, two CpG islands in the p16 exon-2 and intron-2 are homogenously methylated in HCT116, AGS, and MGC803 cells, hence it is likely the homeostatic maintenance of p16 methylation could be observed only in its promoter/exon-1 region in these cells. Because there are many hemi-methylated regions present in the genome, such as the X-chromosomes of female cells and imprinted genes, further study as to whether focal, transient methylation and demethylation are general phenomena in homeostatic maintenance of DNA methylation is certainly warranted.

5hmC plays a crucial role in active DNA demethylation as an intermediate in serial methylcytosine oxidation. However, a certain proportion of 5hmC is not further oxidized, but is instead maintained in the genome of adult tissues such as the brain and colon. Although the additional functions of 5hmC in the genome remain unknown, it is likely to serve an important purpose. It has been reported that hydroxymethylation at both promoter and intragenic locations correlated positively with gene expression [40]. Generally, CpG islands are not methylated in regions around transcription start sites in actively transcribed genes, but are methylated in the gene body. Recently, it has been reported that hydroxymethylation in the genome of the brain is TET2-dependent and more hydroxymethylation is detected in the sense-strands than the antisense-strands of actively transcribed gene bodies [41], [42]. In the present study, we found that the antisense-strands of HCT116 cells contained a certain proportion of “methylated” wildtype p16 alleles that were actually completely hydroxymethylated; but the corresponding sense-strands were not hydroxymethylated. It indicates that hydroxymethylation around the transcription start site in the completely methylated p16 wildtype alleles may be an antisense-strand-specific phenomenon. Further analysis revealed that the asymmetrically methylated and hydroxymethylated (M∶H) wildtype p16 alleles were rendered transcriptionally inactive. It is needed to study whether hydroxymethylation bias toward the antisense-strand is a universal event for all promoter/exon-1 regions in the genome where hydroxymethylation occurs.

While considerably more research will have to be performed to fully elucidate the fine details behind genomic methylation status maintenance, we successfully identified and characterized key differences in inheritance patterns between complete- and hemi-methylated-p16 alleles in both fusion and colon cancer cell lines. In conclusion, the current study illustrated that that the states of completely methylated and unmethylated-p16 alleles are homeostatically maintained in differentiation static cancer cells with concomitant transient focal de novo methylation, hydroxymethylation, and demethylation.

## Supporting Information

Figure S1 **DHPLC characterization of fusion monoclones using two microsatellite markers located within the MTAP-p16/Arf/p15 locus at chromosomal region 9q21.** (**A**) The genotypes of the fusion clones differ from their parental cells at both D9S1749 and D9S974. (**B**) Analysis of chromosome mode for the fusion monoclone-E10 and their parental cells. The number of cells containing a different number of chromosomes is displayed on the left. The chromosome mode was 92 for the fusion clone, 58 for MGC803 cells, and 46 for AGS cells.
(TIF)

Figure S2 **Effect of 5-aza-CdR (5 µM) or TSA (0.1 µM) alone and in combination on proliferation of the fusion and parental cells.** Both the fusion cells and MGC803 cells are very sensitive to the inhibitory effects of TSA, while the AGS cells showed lower sensitivity.
(TIF)

Figure S3 **Bisulfite sequencing of** ***p16*** **alleles in the fusion subclone cells and their parental cells.** Seeding methylation-specific PCR (sMSP) analysis of the 588 bp fragments of the *p16* CpG islands methylated at one of the three seeding sites in intron-1 (26). Each row represents a *p16* molecule; CpG sites (black bar); methylated CpG sites (•); unmethylated CpG sites (**○**); genotype *p16* alleles at SNP rs36228836 (**A** or **T**); focally *de novo* methylated *p16* molecules in the sub-subclone E3 (red arrows).
(TIF)

Figure S4 **Characterization of the methylation states of CpG islands in the** ***p16*** **exon-2 and intron-2.** (**A** and **B**) DHPLC analysis reveals that the exon-2 and intron-2 CpG islands are completely methylated in two representative fusion clones, their parental MGC803 and AGS cells, and HCT116 cells. (**C**) Bisulfite-sequencing shows that the intron-2 CpG islands are homogenously methylated in these cells. (**D**) Locations of three CpG islands within the *p16* allele are illustrated.
(TIF)
