# Supplementary figures and images for: Homeostatic Maintenance of Allele-Specific p16 Methylation in Cancer Cells Accompanied by Dynamic Focal Methylation and Hydroxymethylation
Source: PLoS One. 2014 May 14;9(5):e97785. doi: 10.1371/journal.pone.0097785 (PMC4020935; doi:10.1371/journal.pone.0097785)

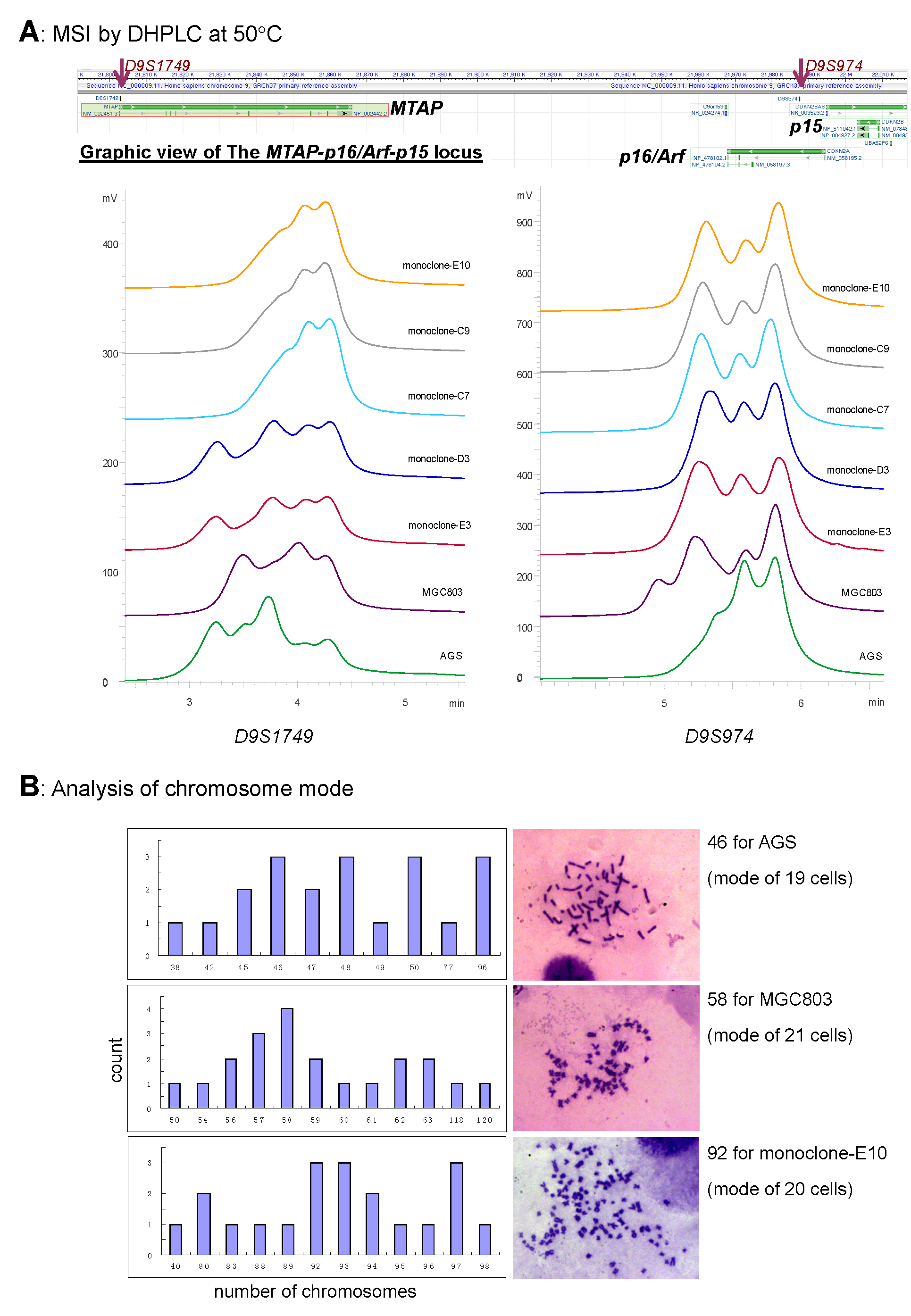

Supplement: Figure S1 — DHPLC characterization of fusion monoclones using two microsatellite markers located within the MTAP-p16/Arf/p15 locus at chromosomal region 9q21. (A) The genotypes of the fusion clones differ from their parental cells at both D9S1749 and D9S974. (B) Analysis of chromosome mode for the fusion monoclone-E10 and their parental cells. The number of cells containing a different number of chromosomes is displayed on the left. The chromosome mode was 92 for the fusion clone, 58 for MGC803 cells, and 46 for AGS cells. (TIF) [file pone.0097785.s001.tif]

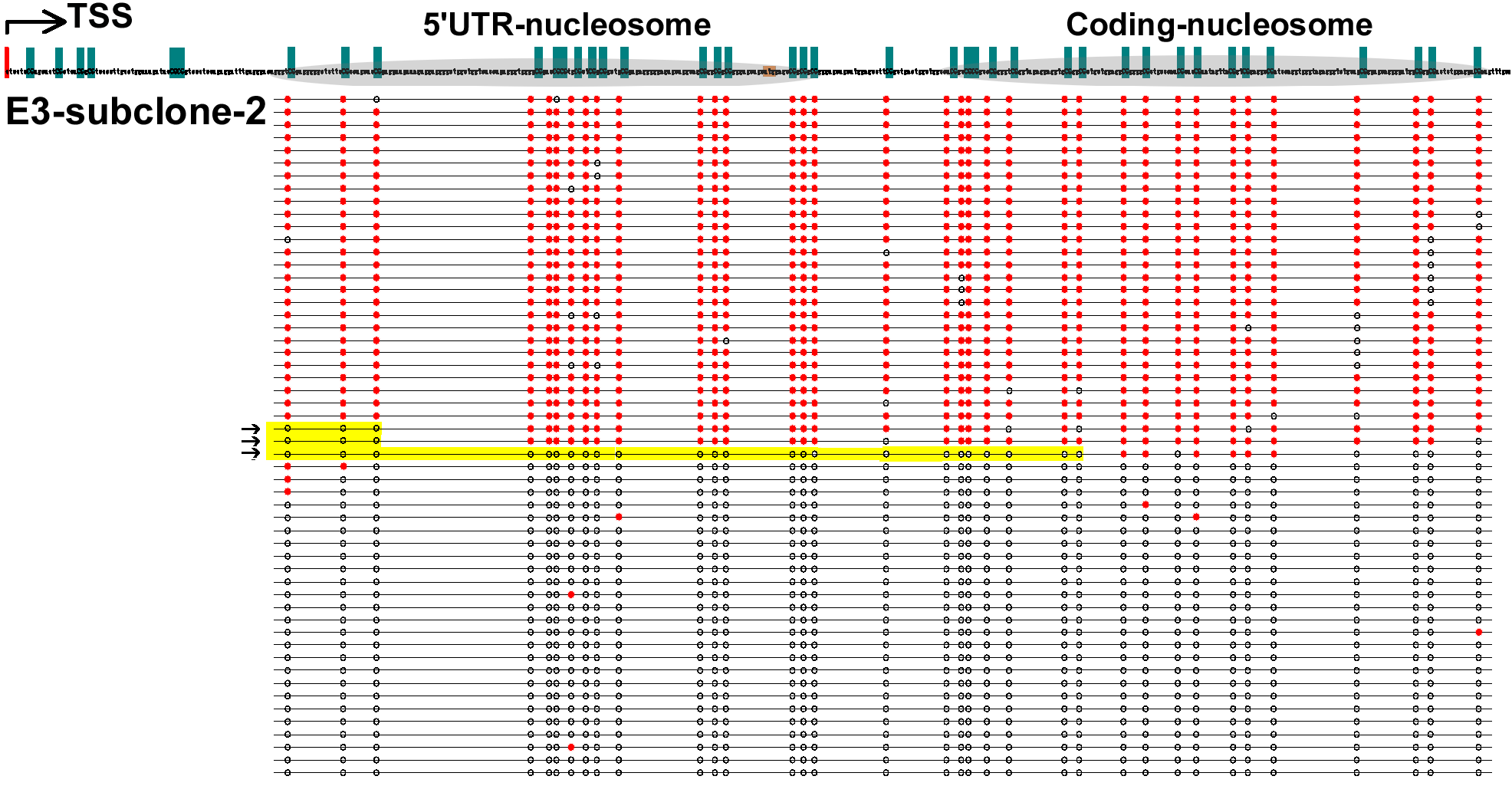

Supplement: Figure S2 — Effect of 5-aza-CdR (5 µM) or TSA (0.1 µM) alone and in combination on proliferation of the fusion and parental cells. Both the fusion cells and MGC803 cells are very sensitive to the inhibitory effects of TSA, while the AGS cells showed lower sensitivity. (TIF) [file pone.0097785.s002.tif]

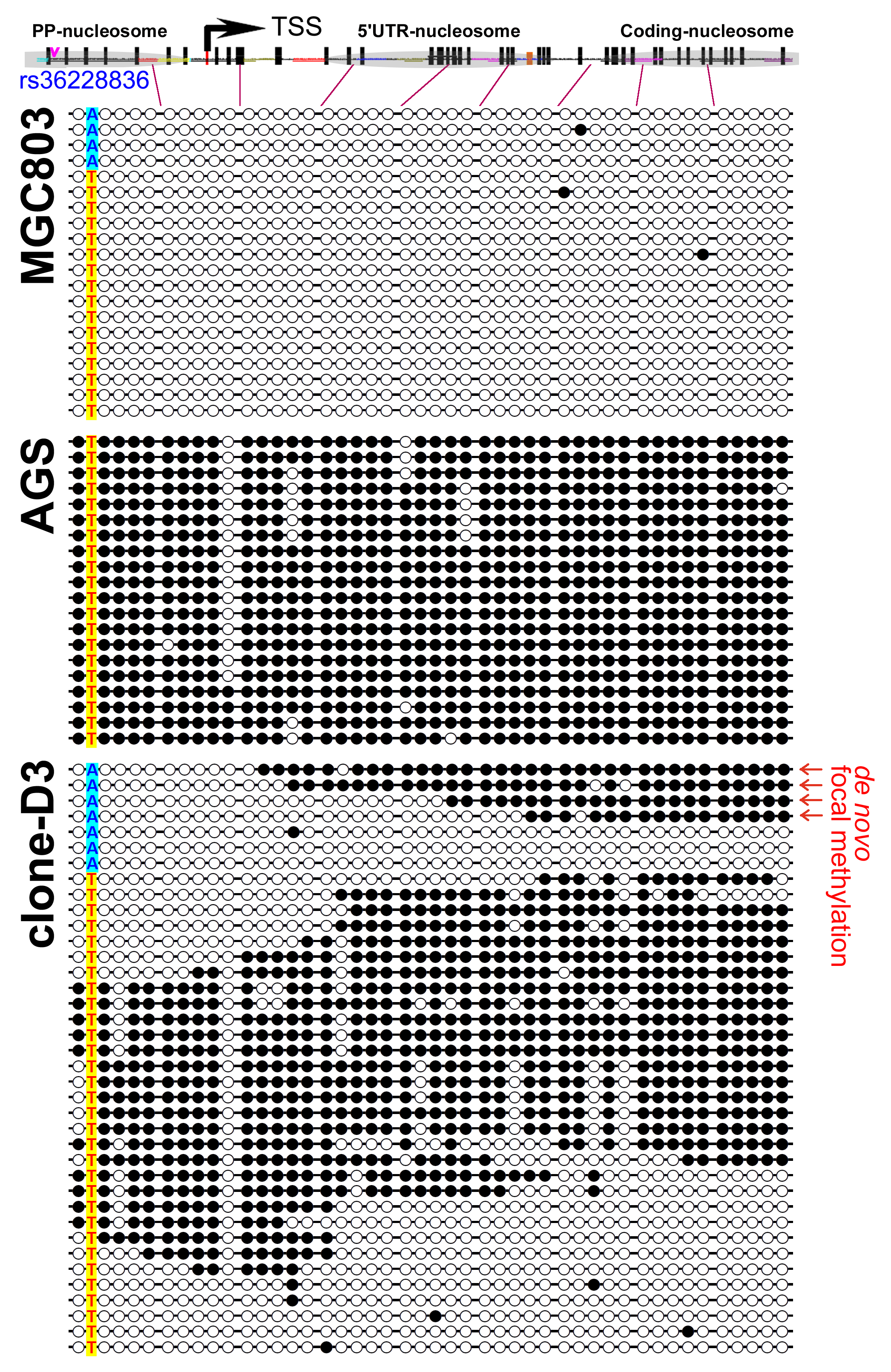

Supplement: Figure S3 — Bisulfite sequencing of p16 alleles in the fusion subclone cells and their parental cells. Seeding methylation-specific PCR (sMSP) analysis of the 588 bp fragments of the p16 CpG islands methylated at one of the three seeding sites in intron-1 (26). Each row represents a p16 molecule; CpG sites (black bar); methylated CpG sites (•); unmethylated CpG sites (○); genotype p16 alleles at SNP rs36228836 (A or T); focally de novo methylated p16 molecules in the sub-subclone E3 (red arrows). (TIF) [file pone.0097785.s003.tif]

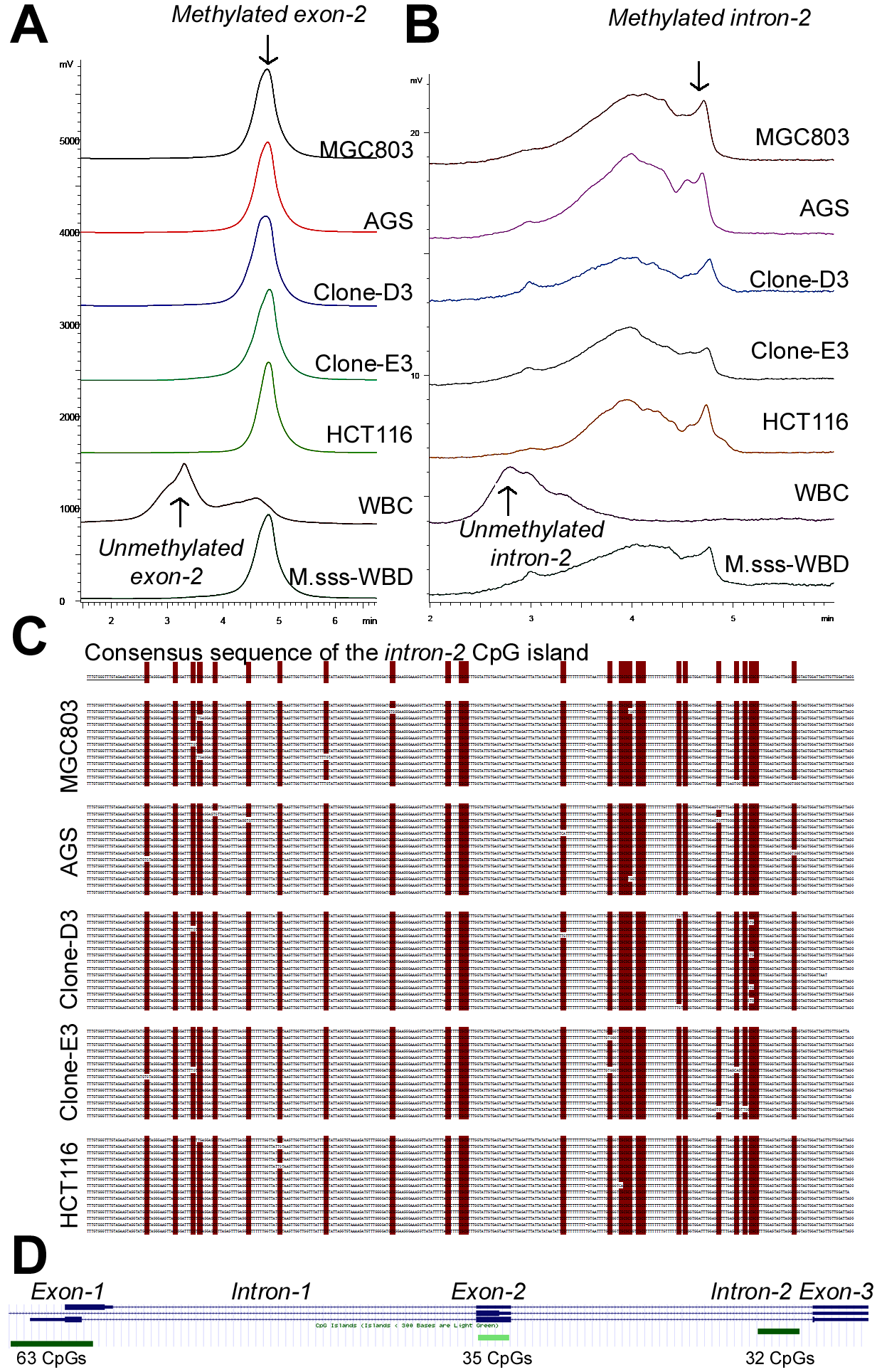

Supplement: Figure S4 — Characterization of the methylation states of CpG islands in the p16 exon-2 and intron-2. (A and B) DHPLC analysis reveals that the exon-2 and intron-2 CpG islands are completely methylated in two representative fusion clones, their parental MGC803 and AGS cells, and HCT116 cells. (C) Bisulfite-sequencing shows that the intron-2 CpG islands are homogenously methylated in these cells. (D) Locations of three CpG islands within the p16 allele are illustrated. (TIF) [file pone.0097785.s004.tif]
